# 

**Published:** 1918-02

**Authors:** 


					﻿PUBLISHED MONTHLY.
SUBSCWJPriON >1.00
ATLANTA
JOURNAL-RECORD
OF ME ICINE
I Atlanta Medical and Surgical Journal. Established 1855. i emi \t
Successor to	041 H YP3r
I and Southern Medical Record, Established 1870.	\ Vrllll I CGI
Vol. LXIV	Atlanta, Ga., February, 1918	No. 11
				

